# 18-24-month HIV-free survival as measurement of the effectiveness of prevention of mother-to-child transmission in the context of lifelong antiretroviral therapy: Results of a community-based survey 

**DOI:** 10.1371/journal.pone.0237409

**Published:** 2020-10-01

**Authors:** Appolinaire Tiam, Michelle M. Gill, Rhoderick Machekano, Vincent Tukei, Majoalane Mokone, Shannon Viana, Mosilinyane Letsie, Mots’oane Tsietso, Irene Seipati, Cecilia Khachane, Marethabile Nei, Florence Mohai, Thorkild Tylleskär, Laura Guay

**Affiliations:** 1 Centre for International Health, University of Bergen, Bergen, Norway; 2 Elizabeth Glaser Pediatric AIDS Foundation, Washington D.C., United State of America; 3 Elizabeth Glaser Pediatric AIDS Foundation, Maseru, Lesotho; 4 Ministry of Health, Maseru, Lesotho; 5 Milken Institute School of Public Health, The George Washington University, Washington D.C., United States of America; University of Ghana College of Health Sciences, GHANA

## Abstract

**Introduction:**

Population-based HIV-free survival at 18–24 months of age among HIV-exposed infants in high prevalence settings in the era of treatment for all is largely unknown. We conducted a community-based survey to determine outcomes of HIV-exposed infants at 18–24 months in Lesotho.

**Methods:**

Between November 2015 and December 2016, we conducted a survey among households with a child born 18–24 months prior to data collection. Catchment areas from 25 health facilities in Butha-Buthe, Maseru, Mohale’s Hoek and Thaba-Tseka districts were randomly selected using probability proportional to size sampling. Consecutive households were visited and eligible consenting caregivers and children were enrolled.

Rapid HIV antibody testing was performed on mothers of unknown HIV status (never tested or tested HIV-negative >3 months prior) and their children, and to children born to known HIV-positive mothers. Information on demographics, health-seeking behavior, HIV, and mortality were captured for mothers and children, including those who died.

The difference in survival between subgroups was determined using the log-rank test.

**Results:**

Of the 1,852 mothers/caregivers enrolled, 570 mothers were HIV-positive. The mother-to-child HIV transmission rate was 5.7% [95% CI: 4.0–8.0]. The mortality rate was 2.6% [95% CI: 1.6–4.2] among HIV-exposed children compared to 1.4% (95% CI: 0.9–2.3) among HIV-unexposed children. HIV-free survival was 91.8% [95% CI: 89.2–93.8] among HIV-exposed infants. Disclosure of mother’s HIV status (aOR = 4.9, 95% CI: 1.3–18.2) and initiation of cotrimoxazole prophylaxis in the child (aOR = 3.9, 95% CI: 1.2–12.6) were independently associated with increased HIV-free survival while child growth problems (aOR = 0.2, 95% CI: 0.09–0.5) were independently associated with reduced HIV-free survival.

**Conclusion:**

Even in the context of lifelong antiretroviral therapy among pregnant and breastfeeding women, HIV has a significant effect on survival among HIV-exposed children compared to unexposed children. Lesotho has not reached elimination of HIV transmission from mother to child.

## Introduction

HIV-free survival is the gold standard in measuring the effectiveness of prevention of mother-to-child HIV transmission (PMTCT) programs especially among breastfeeding populations [1, 2].

Since 2010, new HIV infections among children have declined by 35%, from 270,000 [170,000–400,000] in 2010 to 160,000 [110,000–260,000] in 2018, demonstrating a nearly 64% decline since 2001when almost half a million of children were newly infected with HIV [3]. Most of the infections among children remain attributable to mother-to-child transmission (MTCT) [4, 5]. Program data are now starting to emerge, demonstrating the effectiveness of these approaches in preventing MTCT using real world data which strengthen global reports that use modelling data [2, 6–8]. Most of these studies report HIV-free survival before lifelong antiretroviral therapy (ART) was rolled out.

With an estimated HIV prevalence of 25.6%, Lesotho has the second-highest national HIV prevalence worldwide [9, 10]. Among antenatal care (ANC) attendees, HIV prevalence ranges from 5.4% among adolescents aged 15–19 years, to 21.5% among women aged 20–24 years, 37.5% among those aged 25–29 years, and 40% among those aged 30–39 years [11].

Lesotho’s national guidelines recommend that opt-out HIV testing be offered to all pregnant women presenting to ANC clinic or in labor with unknown HIV status. If HIV-negative, women should be retested for HIV at 36 weeks gestation if the prior test was performed ≥ 6 weeks earlier, or if not done prior to delivery, then HIV testing should be done during labor and delivery [12]. HIV deoxyribonucleic acid polymerase chain reaction (DNA-PCR) or total nucleic acid (TNA) testing is recommended for infants younger than nine months of age in Lesotho. Infants with a positive HIV DNA-PCR or TNA test result are initiated on ART immediately while awaiting results of the repeat confirmatory DNA-PCR test. Final documentation of the child’s HIV status is conducted six weeks following cessation of breastfeeding using HIV DNA-PCR testing among infants younger than nine months of age, or HIV rapid testing, with DNA-PCR if the rapid test is positive, among infants and children older than nine months of age [12].

Traditionally, MTCT rates are assessed at health facility level and focused on six-week MTCT, though in breastfeeding populations, children are exposed to HIV through breastmilk for a prolonged period of time [13]. Facility-based HIV-free survival rate estimates are potentially biased because they exclude women and children who do not utilize health facilities especially in populations where formal health care utilization rates are low. Community-based studies to estimate HIV-free survival among HIV-exposed children overcome some bias concerns in facility-based studies. However, since 2013, when the Lesotho Ministry of Health (MOH) adopted the World Health Organization PMTCT guidelines on universal treatment [8], no community-based assessment of HIV-free survival has been undertaken in Lesotho. The Lesotho population-based impact assessment survey found an overall MTCT rate of 2.8% [14, 15]. We carried out a community household survey to estimate the HIV-free survival of HIV-exposed children 18–24 months of age after the introduction of lifelong ART.

## Materials and methods

### Study design

Between November 2015 and December 2016, we conducted a cross-sectional, community-based survey among households with children born in the previous 18–24 months to estimate MTCT rates, mortality, HIV prevalence, and HIV-free survival among children.

### Sampling procedures

Four districts were purposively selected for the study: Butha-Buthe, Maseru, Mohale’s Hoek, and Thaba Tseka. The first three districts represent the three national ecological zones: highlands, foothills and lowlands. The ecological zones account for variation in overall PMTCT service delivery coverage and heterogeneity in health-seeking behavior due to changing terrain. Maseru was included as the most populous district in the country. The selection of these districts was expected to make the sampling nationally representative. Of the 78 facilities across the four districts, 16 were excluded from the sampling frame, including facilities that were determined to be difficult to reach (e.g., typically only accessible by helicopter). Within each district, we used a multi-stage sampling approach to obtain a representative sample of HIV-positive and HIV-negative mothers and children. First, health facilities from each study district were randomly selected using probability proportional to size sampling based on the annual number of live births. Ten facilities from Maseru District and five facilities each from the other three districts were randomly selected, for a total of 25 facilities. Communities were defined as the villages within the catchment area of a health facility. In each catchment area, all villages were assigned a number and then random selection was used to indicate the village from which recruitment would begin. Recruitment continued according to a pre-determined direction in the next closest village until the approximated sample size target was obtained. Study teams recruited participants from all households meeting the inclusion criteria within a village. If the study team reached approximately half of the target for that catchment area in one village, the team then stopped recruitment and moved to the next village to ensure at least two villages were captured for each catchment area.

To determine the sample size per facility catchment area, we estimated the expected number of HIV-infected women who had given birth in each area proportionate to the catchment population size. To identify eligible households within the catchment area, we went door-to-door or engaged community health workers to identify households in which a mother had delivered a child 18–24 months prior to data collection. If the mother or a primary caregiver was not available to complete the survey, a return appointment was made.

#### Recruitment and enrollment of study population

The study population included mothers and children who were born 18–24 months prior to the data collection time. Household visits were made by trained research assistants responsible for consenting and data collection, and HIV counselors responsible for HIV testing. All eligible households were included in the study if the primary caregiver was willing to provide verbal informed consent. Households were eligible for study participation, regardless of the vital status of the child (alive/deceased). Households were excluded if members of household were not willing to participate, or if the primary caregiver was less than 18 years and was not the father or mother of the child. Households were visited consecutively, informed about the study, and caregivers were invited to participate in the study. If the mother was deceased or was not currently living in the household, consent for study participation was obtained from the child’s primary caregiver.

### Data collection

Caregivers or parents were interviewed in Sesotho or English by research assistants using a structured data collection tool with responses captured electronically. The questionnaire was developed specifically for the purpose of this study and collected data needed to answer study questions. Research assistants first determined the child’s HIV exposure status and then administered the questionnaire to the mother or caregiver. Information collected from the questionnaire included demographics, use of health facilities for HIV and maternal and child health services, maternal HIV status, maternal and infant receipt and use of antiretroviral drugs during pregnancy and after delivery (if known HIV-positive), infant feeding practices, and general well-being, including growth and development. The child health card and/or maternal health card were used to confirm caregiver information. Mortality information for mothers and children who died was also captured, and attempts were made to determine HIV status prior to death.

#### Biologic measurements

During household visits, blood was collected through finger stick from study participants by a trained community counselor for HIV rapid antibody testing according to the national HIV program testing algorithm. The first test performed was the Determine™ HIV-1/2 (manufactured by Alere). Participants with a negative Determine™ assay were told their HIV negative status. All participants who tested positive underwent a second test (with a new finger prick sample) using Uni-Gold™ Recombigen® HIV-1/2 and were told their HIV positive status if the second test was also positive. All maternal participants had a DBS sample taken to be stored in the laboratory for quality control. In addition, DNA-PCR testing was performed on the DBS sample for all maternal participants to confirm the status recorded from the field. Further, DBS specimens were collected from all enrolled children for HIV DNA-PCR confirmatory testing regardless of rapid test results.

All tests conducted for this study were recorded in a log at the testing laboratory using the unique study identification number. Study laboratory test results were obtained directly from the laboratory and entered into the study database to link questionnaire responses with laboratory results. Study data collectors and investigators reviewed laboratory results and identified discordant or confirmatory results that required follow-up by the HIV counselors.

### Sample size calculation and data analysis

Based on previous PMTCT evaluation studies in Rwanda and Malawi, we expected HIV-free survival among 18-24-month-old HIV-exposed infants would be lower than the estimate from Rwanda (91.1%) due to less extensive PMTCT coverage in Lesotho in comparison to Rwanda [16]. Assuming HIV-free survival in Lesotho was about 85%, we needed 545 HIV-exposed infants to estimate HIV-free survival with ±3% precision. Adjusting the sample size by 10% for miscellaneous events (e.g., missing data, consent to some but not all study activities) we targeted to enroll 600 HIV-exposed infants. Given the HIV prevalence of approximately 25.4% among pregnant women in ANC, we would need to visit or approach 2,363 households with children in the 18-24-month age range to identify 600 HIV-exposed infants. To account for potentially 15% refusal rate to participate in the study and interviews with non-maternal caregivers of HIV-exposed children (who may not be able to adequately answer questions needed to address the research aims) we needed to visit approximately 2,700 households to reach the required sample of HIV-exposed infants and their mothers.

We summarized categorical variables using frequencies and percentages. Continuous variables were summarized using means and standard deviations, or medians and interquartile ranges as appropriate. We estimated HIV-free survival as the proportion of children alive and HIV negative among all HIV-exposed children. The precision around survival estimates was summarized using 95% confidence intervals. We used the log-rank test to determine whether there were differences in HIV-free survival between subgroups. Comparisons included HIV-infected versus HIV-exposed but uninfected children; and HIV-exposed versus HIV-unexposed children. Type I error rate (α) for statistical tests was set at 0.05. We performed complete case analysis, and missing data were not imputed.

We used multivariate logistic regression models to test the independent association of ANC attendance, mode and place of delivery, gestational age at birth, birth weight, maternal vital status, adherence to and timing of initiation of antiretroviral drugs, infant feeding method and nutritional status with HIV-free survival among HIV-exposed children. All statistical data analysis was performed using STATA version 14.2 (Stata Corp).

### Ethical considerations

The study was approved by the Lesotho MOH Research and Ethics Committee and the George Washington University’s Office of Human Research. All participants were informed of the study objectives, were given the opportunity to ask questions and provided verbal informed consent to participate in the study.

Once a household was determined to meet the eligibility criteria, the mother/caregiver of the eligible child was identified and informed about the evaluation. The verbal informed consent text included statements that described the purpose of the evaluation, the activities to be conducted and confidentiality protections, in addition to a specific statement for mothers/caregivers to provide consent for themselves and their children (if applicable). The interviewer read each of these statements in the participants’ language of choice (English or Sesotho) exactly as written and documented the response on an electronic device used for data collection with a password verification process. The mother/caregiver had the opportunity to ask questions regarding enrollment into the evaluation. The consent process was structured so that a participant could agree to participate in some activities, but not others. Biological mothers were the preferred participants, but if the mother was deceased or not currently living in the household, consent was obtained from the child’s primary caregiver, who was asked to respond to questions to the extent possible.

## Results

**Fig 1:**
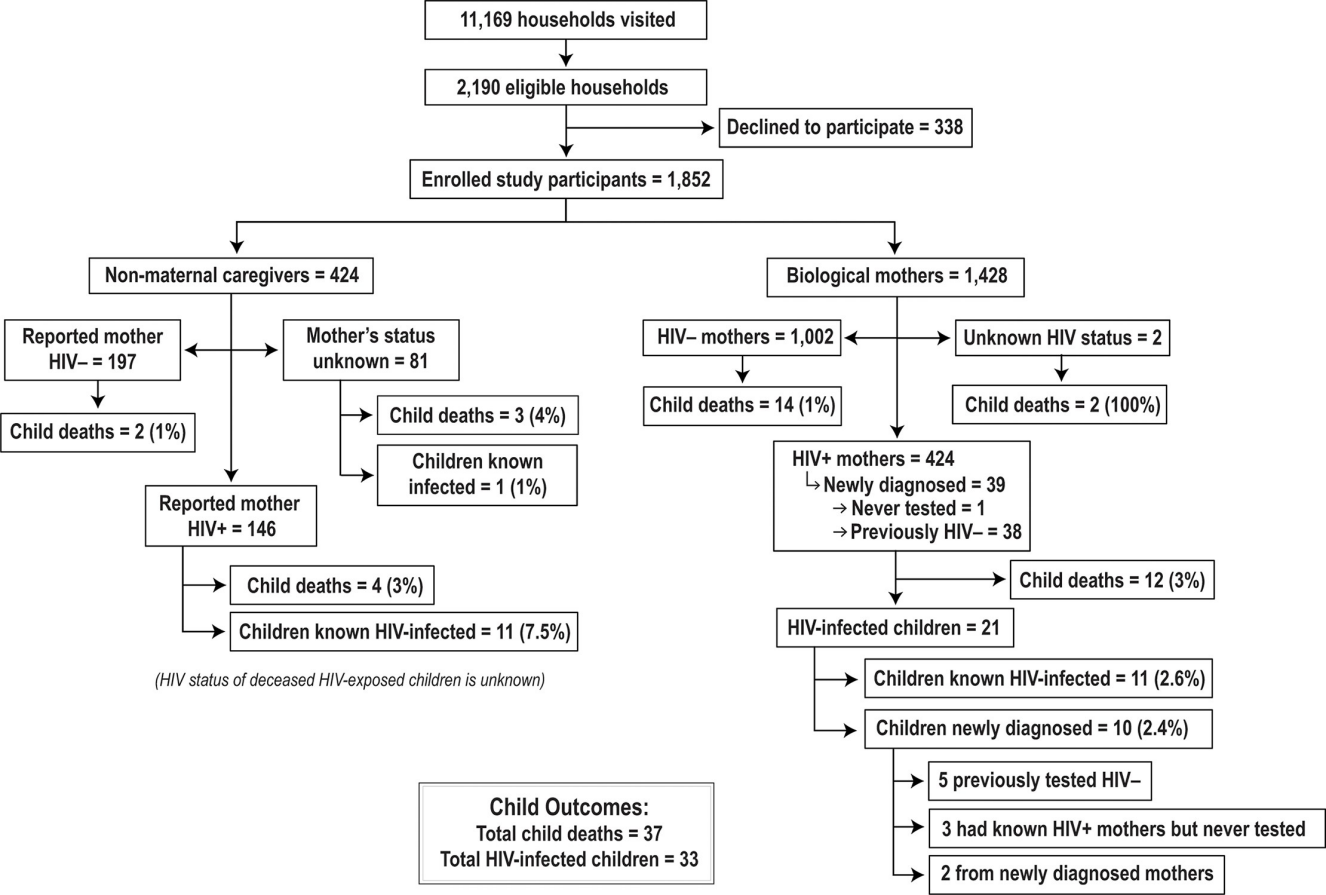
Participants screening, enrollment, and outcomes.

Between November 2015 and December 2016, we screened 11,169 households for the study eligibility (Fig 1). Of the 2,190 eligible households, 1,852 mothers/caregivers consented and were enrolled in the study. Of the 338 households that declined to participate, the following reasons were provided: caregiver, child or both were unavailable (n = 178), (157 of whom were at work); lack of time to participate (n = 71); no incentive provided (n = 25); not comfortable to participate (n = 16); caregiver did not feel they had enough information to answer questions (n = 15); mother needed husband’s permission (n = 9); caregiver was below 18 years of age (n = 6); and other reasons (n = 18).From the enrolled households, there were 1,884 deliveries (including 66 twin births) that occurred 18–24 months prior to data collection. Most of the children (77.1%, n = 1,428) were being cared for by their biological mothers. Nearly a third (30.7%) of biological mothers reported and knew their HIV-positive status. Thirty-nine mothers were newly diagnosed HIV-positive during the study, of whom 38 had previously tested HIV-negative during the index pregnancy. A total of 1,199 (64.7%) biological mothers were HIV-negative and 83 mothers were of unknown HIV status and unavailable for testing.

### Characteristics of caregivers

The mean age (±SD) of mothers was 27.8 ± 6.1 years (Table 1). Overall, 95.8% of mothers attended ANC at least once with over 80% of women delivering in health facilities. One-fifth (20.8%) of mothers presented with known HIV status at ANC. Most HIV-positive mothers (94.7%) disclosed their HIV status with 59.9% disclosing it to their spouse or partner.

**Table 1:** Antenatal, delivery and HIV service utilization by study participants.

	HIV-negative Mothers (N = 1199) n (%)	HIV-positive Mothers (N = 570) n (%)	Mothers with Unknown HIV Status (N = 83) n (%)	Total (N = 1852) n (%)
Mean Age (SD)	26.7 (5.9)	30.1 (6.0)	27.7 (5.2)	27.8 (6.1)
Attended ANC during pregnancy	1152 (96.4)	530 (94.8)	56 (93.3)	1738 (95.8)
Mean gestational age in weeks at first ANC (SD)	16.7 (7.5)	16.6 (7.9)	14.7 (8.1)	16.7 (7.6)
Attended 4+ ANC visits	710 (70.6)	302 (68.8)	7 (53.8)	1019 (70.0)
Child delivered at a health facility	934 (81.1)	416 (78.5)	45 (83.3)	1395 (80.4)
Mother known HIV-positive during pregnancy	-	114 (20.8)	-	114 (6.6)
Mother tested for HIV during pregnancy	1161 (98.1)	418 (73.3)	8 (80.0)	1587 (85.7)
Mother disclosed HIV status to anyone	918 (79.7)	514 (94.7)	-	1432 (84.3)
Mother disclosed HIV status to spouse/partner	706 (76.9)	308 (59.9)	-	1014 (70.8)
Mother disclosed HIV status to other family members	362 (39.4)	373 (72.6)	-	735 (51.3)
Mother disclosed HIV status to others	4 (0.4)	26 (5.1)	-	49 (3.4)

### Characteristics of children

The child participants included 64 twins (3.4%) and 52.5% of the children were male (Table 2). Preterm births occurred in 9.1% of children, with HIV-exposed children slightly more likely to have a preterm birth. Overall, 95.2% of infants were ever breastfed, with a breastfeeding rate of 90.7% among HIV-exposed infants. The mean (SD) birth weight was 3.0 (0.5) with HIV exposed infants having slightly lower mean birth weight compared to HIV unexposed infants. HIV-unexposed infants were breastfed longer with a mean of 15 months compared to HIV-exposed infants, who were breastfed for a mean of 11 months.

**Table 2:** Characteristics of children by HIV exposure status.

	HIV-unexposed children (n = 1207) n (%)	HIV-exposed children (n = 582) n (%)	Children with unknown HIV exposure (n = 95) n (%)	Total (N = 1884)
Twin	34 (2.8)	30 (5.1)	0 (0)	64 (3.4)
Male	609 (50.5)	324 (55.7)	56 (59.0)	989 (52.5)
Stillbirth	4 (0.3)	2 (0.3)	2 (2.1)	8 (0.4)
Preterm birth	106 (9.0)	54 (9.9)	3 (3.6)	163 (9.1)
Mean birth weight (SD)	3.05 (0.5)	2.9 (0.6)	3.06 (0.4)	3.02 (0.5)
Child ever breastfed	1169 (97.6)	519 (90.7)	81 (91.0)	1769 (95.2)
Child still breastfeeding after 18 months	200 (17.1)	47 (9.1)	3 (3.7)	250 (14.1)
Median time child was breastfed (IQR) (months)	15 (10–18)	11 (6–14)	12 (6–16)	12 (7–18)

There were 37 reported child deaths, 16 of whom were from HIV-positive mothers. Thirty-three children were identified as HIV-positive within the participating households among whom 10 were newly diagnosed during the study. Of the ten children who were newly diagnosed with HIV, five had previously tested HIV-negative, three were from known HIV-positive mothers but had never been tested, and two were from mothers who were newly diagnosed during the study.

#### Maternal use of antiretroviral therapy

Overall, 86.8% of HIV-positive women used ART during pregnancy. Based on child HIV status, 87.4% and 71.4% of mothers of HIV-uninfected children and HIV-infected children were enrolled on ART respectively (Table 3). Most mothers (89.1%) initiated ART either before or during pregnancy. Among mothers of HIV-infected children, 37.6% started ART either during or after breastfeeding period compared to 9.6% of mothers of HIV-uninfected children. At the time of the study, 88.0% of HIV-positive mothers reported currently taking ART. Among HIV-exposed children, 89.3% received nevirapine prophylaxis within three days of delivery. Of the 13 HIV-infected children who had records about their treatment initiation, 12 were initiated on ART.

**Table 3:** Maternal use of antiretroviral drugs during pregnancy by child’s HIV status, excluding caregivers.

	Uninfected, HIV-exposed children (N = 367) n (%)	Infected, HIV-exposed children (N = 19) n (%)	Total HIV-exposed children (N = 396)
Mother had a CD4 test done when pregnant	282 (83.7)	12 (75.0)	294 (85.3)
Missing	30	3	33
Mother received ART during this pregnancy	285 (87.4)	10 (71.4)	295 (86.8)
Missing	41	5	46
Medicine received by mothers who took antiretroviral drugs during pregnancy*			
AZT	16 (5.7)	0	16 (5.5)
ART for life	261 (92.9)	10 (100.0)	271 (93.1)
Other	4 (1.4)	0	4 (1.4)
Missing	4	0	4
Time mothers started taking ART*			
Before pregnancy with this child	131 (39.5)	4 (25.0)	135 (38.8)
During pregnancy with this child	169 (50.9)	6 (37.5)	175 (50.3)
During breastfeeding of this child	20 (6.0)	5 (31.3)	25 (7.2)
After breastfeeding of this child	12 (3.6)	1 (6.3)	13 (3.7)
Missing	35	3	38
Regimen taken by the mother during pregnancy†			
TDF+3TC+EFV	204 (80.3)	8 (80.0)	212 (80.3)
TDF+3TC+NVP	16 (6.3)	2 (20.0)	18 (6.8)
AZT+3TC+EFV	10 (3.9)	0	10 (3.8)
AZT+3TC+NVP	21 (8.3)	0	21 (8.0)
Other	3 (1.2)	0	3 (1.1)
Missing	7	0	7
Mother currently taking ART	313 (88.2)	16 (84.2)	329 (88.0)

* Total N is among those who received antiretroviral drugs during pregnancy

†Total N is among those who received ART for life

### Mortality, HIV transmission, and HIV-free survival among children

Tables 4 and 5 summarize child outcomes by HIV exposure status. The estimated child mortality was 2.6% (95% CI: 1.6–4.2) among HIV-exposed children compared to 1.4% (95% CI: 0.9–2.3) among HIV-unexposed children. The estimated HIV transmission was 5.7% (95% CI: 4.0–8.0). HIV-free survival was 91.8% (95% CI: 89.2–93.8). The majority of child deaths occurred after the neonatal period (Table 5).

**Table 4:** Child mortality and HIV-free survival by HIV status.

Child Outcomes	HIV-unexposed Children % [95% CI]	HIV-exposed Children % [95% CI]	Total Children % [95% CI]
Child mortality	1.4 [0.9–2.3]	2.6 [1.6–4.2]	1.8 [1.3–2.5]
HIV infection	-	5.7 [4.0–8.0]	-
HIV-free survival	-	91.8 [89.2–93.8]	-

**Table 5:** Timing of children’s death by HIV exposure (N = 28).

Timing of death	HIV-unexposed children n (%)	HIV-exposed children n (%)	Children with unknown status n (%)	Total Children (N = 28)
Day 0	4 (26.7)	0	2 (100)	6 (21.4)
Within 24 hours	1 (6.7)	1 (9.1)	0	2 (7.2)
Within 28 days	3 (20.0)	3 (27.3)	0	6 (21.4)
More than 28 days	7 (46.6)	7 (63.6)	0	14 (50.0)

#### Factors associated with HIV-free survival among children

Table 6 summarizes factors associated with mortality, HIV transmission and HIV-free survival among children. Disclosure of mother’s HIV status (aOR = 4.9, 95% CI: 1.3–18.2) and initiation of cotrimoxazole prophylaxis in child (aOR = 3.9, 95% CI: 1.2–12.6) were independently associated with increased HIV-free survival while child growth problems (aOR = 0.2, 95% CI: 0.09–0.5) was independently associated with reduced HIV-free survival.

**Table 6:** Factors associated with child mortality, HIV transmission and HIV-free survival.

Variable	Unadjusted OR [95% CI]	p-value	Adjusted OR [95% CI]	p-value
**Factors Associated with Infant Mortality**				
Infant was a twin (ref = No)	7.1 [3.0–16.8]	0.00	1.4 [0.2–8.7]	0.70
Mother HIV-positive (ref = Negative)	1.9 [0.9–3.7]	0.09	2.0 [0.6–6.8]	0.25
Child delivered in health facility (ref = No)	0.3 [0.1–0.5]	0.00	0.3 [0.1–1.0]	0.06
Timing of child birth (ref = before time)	0.3 [0.1–0.9]	0.02	0.5 [0.1–2.0]	0.32
Mother told child had a growth problem (ref = No)	4.0 [1.5–10.6]	0.01	2.8 [0.8–9.8]	0.11
Child ever breastfed (ref = No)	0.1 [0.0–0.2]	0.00	0.2 [0.0–0.9]	0.04
Infant breastfed for more than 6 months (ref = No)	0.2 [0.1–0.5]	0.00	0.7 [0.2–2.9]	0.60
**Factors Associated with HIV Infection**				
Disclosed HIV test result (ref = no)	0.3 [0.1–0.9]	0.04	0.3 [0.1–1.7]	0.18
Mother told child had a growth problem (ref = no)	2.6 [1.1–6.4]	0.03	4.6 [1.5–13.5]	0.01
Breastfeeding for 6+ months (ref = no)	2.3 [0.9–5.6]	0.06	2.3 [0.8–6.5]	0.12
Started ART (ref = before BF)				
During pregnancy	1.5 [0.5–4.4]	0.49	1.2 [0.4–3.9]	0.75
During breastfeeding	9.3 [2.6–32.9]	0.00	3.2 [0.6–15.9]	0.16
Not on ART/After breastfeeding	6.3 [1.8–22.2]	0.00	1.9 [0.2–17.0]	0.57
Received ART throughout breast feeding (ref = yes)	0.3 [0.1–0.8]	0.01	2.2 [0.4–12.8]	0.38
Child was given first dose of NVP syrup within 3 days after delivery (ref = no)	0.3 [0.1–0.6]	0.00	0.3 [0.1–1.1]	0.08
Child was given CTX to take daily beginning at 6 weeks (ref = no)	0.2 [0.1–0.6]	0.00	0.4 [0.1–1.5]	0.18
**Factors Associated with HIV-free Survival**				
Disclosed HIV test result (ref = no)	2.9 [1.0–8.1]	0.04	4.9 [1.3–18.2]	0.017
Delivered in a facility (ref = no)	2.3 [1.4–4.7]	0.00	1.7 [0.7–4.2]	0.22
Mother told child had a growth problem (ref = no)	0.2 [0.1–0.5]	0.00	0.2 [0.1–0.5]	<0.01
When started ART (ref = before BF)				
During pregnancy	0.7 [0.3–1.6]	0.39	0.7 [0.3–1.8]	0.44
During breastfeeding	0.1 [0.0–0.4]	0.00	0.3 [0.1–1.3]	0.11
Not on ART/After breastfeeding	0.2 [0.1–0.6]	0.01	0.7 [0.1–4.2]	0.69
Received ART throughout breast-feeding (ref = yes)	2.8 [1.3–5.9]	0.01	0.4 [0.1–1.8]	0.26
Child was given first dose of NVP syrup within 3 days after delivery (ref = no)	4.0 [2.2–7.5]	0.00	2.1 [0.6–7.0]	0.22
Child was given CTX to take daily beginning at 6 weeks (ref = no)	5.4 [2.7–10.8]	0.00	3.9 [1.2–12.6]	0.02

## Discussion

We found that HIV-free survival in Lesotho was 91.8%. Our results demonstrate the effectiveness of the Lesotho PMTCT program in averting HIV infection and deaths in children within the first 18–24 months of life after introduction of lifelong ART for all HIV-positive pregnant women in 2013. Our finding corroborates with a systematic review of 18 studies published between 2005 and 2015, that estimated HIV-free survival in a breastfed population found that 18-month HIV-free survival estimates were 89.0% (95% CI 83.9%, 94.2%) with maternal ART for six months (five studies) and 96.1% (95% CI: 92.8%-99.0%) with maternal lifelong ART (three studies) [17]. HIV-free survival at 24 months was 89.2% (95% CI: 79.9%-98.5%) for children whose mothers were on ART for six months (two studies) [17]. Among breastfed infants, HIV-free survival ranged from 87% (95% CI: 78%-92%) to 96% (95% CI: 91%-98%).

Similarly, in a study in Eswatini, that reported HIV-free survival prior to the era of lifelong ART for pregnant and breastfeeding women in that country, HIV-free survival was 95.9% (95% CI: 94.1–97.2) though the authors reported a low death rate due to cultural sensitivities in collecting death data which may have impacted overall HIV-free survival [8].

Although approaching elimination, the MTCT rate estimated in our study was 5.7% [95%CI 4.0–8.0]. In the context of Lesotho, this shows much progress compared to the UNAIDS MTCT rates reported to be 12.7% (9.9–14.4) in 2019, and 30% in 2004 [18]. As UNAIDS estimates are based on models, which make assumptions that may not be accurate, estimates from surveys like ours are more reliable. The current UNAIDS estimates were based on PMTCT coverage of 77% (59–89) and early infant diagnosis coverage of 70% (60–90) (15). Further, assuming all children who died were HIV-infected, the percentage of children who died or were HIV infected was 8.2% [95%CI 6.2–10.8] versus 12.7% (9.9–14.4) for UNAIDS transmission. Although mortality among HIV-infected children is high, at most only 50% of HIV-infected children will die by their second birthday making it unlikely that all children who died were HIV-infected [4–7]. However, much needs to be done to ensure women living with HIV are diagnosed even ahead of their index pregnancy or ideally ahead of their first pregnancy because women who start ART before pregnant and are virally suppressed are less likely to transmit HIV to their babies in utero, perinatally or through breastfeeding. The MTCT rate in Lesotho remains unacceptably high, and over three years after the rollout of lifelong ART for HIV-positive pregnant women, the program has not yet achieved elimination (an MTCT rate of < 5% and 2% in breastfeeding and non-breastfeeding population respectively) [19]. In addition, our study shows differences between MTCT rates reported from clinical trials and real world rates. Former studies have consistently reported MTCT rates lower than 5% [18, 20, 21].

One-third of HIV-infected children were diagnosed during the study. Eight of the ten children have been in contact with health system for various services. However, contrary to the recommendation that all HIV-exposed children should have the final HIV test at 18–24 months or six weeks after cessation of breastfeeding, this was not the case for the eight children [8]. Five out of ten had tested HIV-negative initially but had not had subsequent test, while three children whose mothers were on ART had never been tested. This finding shows a need to look for HIV-infected children beyond PMTCT setting. In a systematic review of 26 papers, Cohn et. al found that HIV prevalence was highest in pediatric inpatient settings (21.1%, 95% CI: 14.9–27.3), nutrition centers (13.1%, 95% CI: 3.4–22.7), immunization centers (3.3%, 95% CI: 0–6.9), then pediatric outpatient (2.7%, 95% CI: 0.3–5.2). Symptom-triggered universal testing in pediatric outpatient settings had a diagnostic yield similar to that found in the inpatient ward (21.3%, 95% CI: 11.6–31.0 in triggered testing vs 20.9%, 95% CI: 13.5–28.3 in universal testing) [22]. In a recent paper, data from Cameroon showed nontraditional PMTCT entry points [adjusted odds ratio (aOR): 1.95; 95% confidence interval (CI): 1.36 to 2.80] was independently associated with HIV positivity among HIV-exposed children [23]. In a high prevalence country like Lesotho, optimizing testing with screening of all HIV-exposed children at 18–24 months who do not have final HIV status will assist in diagnosing more children.

Our study also explored factors associated with MTCT and/or death. Factors associated with MTCT are well elucidated in the literature [22, 24, 25]. Factors associated with HIV-free survival in Lesotho included HIV-positive mothers disclosing their HIV status and HIV-exposed infants taking cotrimoxazole prophylaxis. It can be hypothesized that women who disclosed their HIV status got more support to be adherent to their treatment [26, 27]. HIV-exposed infants who had growth difficulties were more likely to be HIV-infected and/or to die.

The limitations of the study include the fact that information for more than a quarter of children was received from caregivers who were not biological mothers. Although the team took all measures to verify the information in the handheld health records, in some instances such information was not available. However, our results remain valid considering the large sample size, which took these challenges into consideration.

In addition, for several deceased children, we did not know their HIV status and the cause of death was not recorded in any home-kept document. This limitation did not have impact on our results because the study was measuring HIV-free survival and was not set to describe HIV-related deaths.

Although the data collection was completed almost five years ago, the results of this study remain relevant especially because it is the first and so far the only community-based survey carried out in Lesotho to measure HIV-free survival.

## Conclusion

Even in the context of lifelong ART among pregnant and breastfeeding women, HIV has a significant effect on survival among exposed children. HIV transmission to mothers and children continue to occur during breastfeeding period, therefore it is important to test mother-child pairs during the breastfeeding period while they attend routine clinics such as postnatal care, immunization clinics, outpatient and inpatient services. This study has shown that Lesotho has not reach elimination MTCT despite introduction lifelong ART for all HIV-positive women. Since this study measures the effectiveness of PMTCT program when the guidelines were just changed, it is expected that the performance of program will continue to improve to reach the MTCT elimination level.

We recommend that the MOH develop a national registry that links HIV-exposed infants with their mother until final HIV status is ascertained at 18–24 months as per national guidelines. In addition, there is a need to repeat this study and triangulate data to measure impact of increased coverage of PMTCT services across Lesotho.

## Supporting information

S1 Questions (DOCX)

S1 Data (XLSX)

## References

[1] Stringer EM, Chi BH, Chintu N, Creek TL, Ekouevi DK, Coetzee D, et al Monitoring effectiveness of programmes to prevent mother-to-child HIV transmission in lower-income countries. Bull World Health Organ. 2008;86(1):57–62. 10.2471/blt.07.043117 18235891 PMC2647351

[2] Goga AE, Dinh TH, Jackson DJ. Evaluation of the Effectiveness of the National Prevention of Mother-to-Child Transmission (PMTCT) Programme Measured at Six Weeks Postpartum in South Africa, 2010. Cape Town, South Africa: South African Medical Research Council, National Department of Health of South Africa and PEPFAR/US Centers for Disease Control and Prevention; 2012. Available from: https://www.samrc.ac.za/sites/default/files/files/2016-07-12/SAPMTCTE2010.pdf [Accessed May 25, 2020].

[3] Joint United Nations Programme on HIV/AIDS (UNAIDS). Global HIV & AIDS Statistics—2019 fact sheet. Available from: https://www.unaids.org/en/resources/fact-sheet. [Accessed May 25, 2020].

[4] Idele P, Hayashi C, Porth T, Mamahit A, Mahy M. Prevention of mother-to-child transmission of HIV and paediatric HIV care and treatment monitoring: from measuring process to impact and elimination of mother-to-child transmission of HIV. AIDS Behav. 2017;21(Suppl 1):23–33. 10.1007/s10461-016-1670-9 28063074 PMC5515952

[5] World Health Organization. Consolidated Guidelines on the Use of Antiretroviral Drugs for Treating and Preventing HIV Infection. Geneva: World Health Organization; 2013. 24716260

[6] Ruton H, Mugwaneza P, Shema N, Lyambabaje A, de Dieu Bizimana J, Tsague L, et al HIV-free survival among nine- to 24-month-old children born to HIV-positive mothers in the Rwandan national PMTCT programme: a community-based household survey. J Int AIDS Soc 2012;15(1):4 10.1186/1758-2652-15-4 22289641 PMC3293013

[7] Joint United Nations Programme on HIV/AIDS (UNAIDS) Start Free Stay Free AIDS Free 2019 report https://www.unaids.org/en/resources/documents/2019/20190722_UNAIDS_SFSFAF_2019 [Accessed July 17, 2020]

[8] Chouraya C, Machekano R, Mthethwa S, Lindan K, Mirira M, Kudiabor K, et al Mother-to-child transmission of HIV and HIV-free survival in Swaziland: A community-based household survey. AIDS Behav. 2018 7;22(Suppl 1):105–113. 10.1007/s10461-018-2121-6 29696404 PMC6045958

[9] Lesotho Ministry of Health. National Guidelines for the Prevention of Mother-to-Child Transmission of HIV, 2013. Maseru: Government of Lesotho; 2013.

[10] Joint United Nations Programme on HIV/AIDS (UNAIDS) Data 2019. Available: https://www.unaids.org/sites/default/files/media_asset/2019-UNAIDS-data_en.pdf [Accessed: May 25, 2020]

[11] Lesotho Ministry of Health and ICF Macro. Lesotho Demographic and Health Survey 2014. Maseru: Ministry of Health; 2016.

[12] Government of Lesotho. The National HIV Testing Services Guidelines. Maseru, Lesotho: Ministry of Health; 2016.

[13] Feucht UD, Meyer A, Kruger M. Missing HIV prevention opportunities in South African children—a 7-year review. BMC Public Health 2014; 14:1265 10.1186/1471-2458-14-1265 25495201 PMC4300827

[14] Bureau of Statistics (Lesotho), Centers for Disease Control and Prevention (CDC), ICAP, Columbia University Mailman School of Public Health, Ministry of Health (Lesotho). Lesotho Population-based Impact Assessment Report 2016–2017. Maseru, Lesotho: Ministry of Health; 2019.

[15] The U.S. President’s Emergency Plan for AIDS Relief. Lesotho Country Operational Plan (COP) 2018: Strategic Direction Summary. Available at: https://www.state.gov/wp-content/uploads/2019/08/Lesotho-2.pdf. [Accessed May 25, 2020].

[16] Mandala J, Moyo T, Torpey K, Weaver M, Suzuki C, Dirks R, et al Use of service data to inform pediatric HIV-free survival following prevention of mother-to-child transmission programs in rural Malawi. BMC Public Health. 2012;12:405 Published 2012 Jun 6. 10.1186/1471-2458-12-405 22672627 PMC3425277

[17] Chikhungu LC, Bispo S, Rollins N, Siegfried N, Newell ML. HIV-free survival at 12–24 months in breastfed infants of HIV-infected women on antiretroviral treatment. Trop Med Int Health. 2016;21(7):820–8. 10.1111/tmi.12710 27120500 PMC5096069

[18] Shapiro RL, Kitch D, Ogwu A, Hughes M Lockman S, Powis K et al HIV transmission and 24-month survival in a randomized trial of HAART to prevent MTCT during pregnancy and breastfeeding in Botswana. AIDS. 2013: 27: 1911–1920. 10.1097/qad.0b013e32836158b0 24180000 PMC3987116

[19] Vrazo AC, Sullivan D, Ryan Phelps B. Eliminating Mother-to-Child Transmission of HIV by 2030: 5 Strategies to Ensure Continued Progress. Glob Health Sci Pract. 2018;6(2):249–256. Available at: https://www.ncbi.nlm.nih.gov/pmc/articles/PMC6024627/ [Accessed May 25, 2020]. 10.9745/GHSP-D-17-00097 29959270 PMC6024627

[20] The Kingdom Of Lesotho, Government of Lesotho. Final Report for a Joint Review of HIV/Tuberculosis and Hepatitis Programmes. Maseru, Lesotho;2017. Available at: http://www.unaids.org/sites/default/files/country/documents/LSO_2018_countryreport.pdf [Accessed: May 25, 2020].

[21] Jamieson DJ, Chasela CS, Hudgens MG, King CC, Kourtis AP, Kayira D, et al Maternal and infant antiretroviral regimens to prevent postnatal HIV-1 transmission: 48-week follow-up of the BAN randomised controlled trial. Lancet 2012: 379: 2449–2458. 10.1016/S0140-6736(12)60321-3 22541418 PMC3661206

[22] Cohn J, Whitehouse K, Tuttle J, Lueck K, Tran T. Paediatric HIV testing beyond the context of prevention of mother-to-child transmission: a systematic review and meta-analysis. Lancet HIV. 2016;3(10):e473–81. 10.1016/S2352-3018(16)30050-9 27658876

[23] Tchendjou P, Nzima V, Lekeumo S, Sacks A, Bianchi F, Lemaire JF, et al HIV Mother-to-Child Transmission in Cameroon: EID Positivity Yields and Key Risk Factors by Health Service Points After Usage of POC EID Systems. J Acquir Immune Defic Syndr. 2020;84 Suppl 1:S34–S40. 32520913 10.1097/QAI.0000000000002374

[24] Newell ML. Prevention of mother-to-child transmission of HIV: challenges for the current decade. Bull World Health Organ. 2001;79(12):1138–1144. 11799446 PMC2566720

[25] Taha TE, Hoover DR, Kumwenda NI, Fiscus SA, Kafulafula G, Nkhomaet C, et al Late postnatal transmission of HIV-1 and associated factors. J Infect Dis. 2007;196:10–4 10.1086/518511 17538877

[26] Gouse H, Henry M, Robbins RN, et al Psychosocial Aspects of ART Counseling: A Comparison of HIV Beliefs and Knowledge in PMTCT and ART-Naïve Women. J Assoc Nurses AIDS Care. 2017;28(4):504–517. 10.1016/j.jana.2017.03.002 28377125 PMC5468486

[27] Kalichman SC, Kalichman MO, Cherry C, and Grebler T. HIV disclosure and transmission risks to sex partners among HIV-positive men. AIDS Patient Care STDS. 2016;30(5):221–8. 10.1089/apc.2015.0333 27158850 PMC4870647

