# Supplementary material for: 18-24-month HIV-free survival as measurement of the effectiveness of prevention of mother-to-child transmission in the context of lifelong antiretroviral therapy: Results of a community-based survey
Source: PLoS One. 2020 Oct 1;15(10):e0237409. doi: 10.1371/journal.pone.0237409 (PMC7529246; doi:10.1371/journal.pone.0237409)
Supplement: S1 Questions — (DOCX) [file pone.0237409.s001.docx]

**INTERVIEW FOR WOMEN/CAREGIVERS**

**Date of Interview: ­___ ___ / ___ ___ / 2 0 1 ___ Interviewer ID:** □□

**Facility Catchment Area: ______________________________**

**Village: _________________________**

**Household ID:** □□□□

*Unless otherwise noted, do not read pre-coded responses and select only one response for questions with multiple options or write in the answer in the space provided.*

**Interviewers to complete the following questions BEFORE the interview:**

| Is there an available antenatal record or bukana for CHILD where his/her mother’s antenatal care is written down? | **Yes=1**  **No=0** |
| --- | --- |
|  |  |
| Was there an available record or bukana for CHILD where his/her under 5 MCH care is written down? | **Yes=1**  **No=0** |
|  |  |

*I would first like to record information about all of your children that were born in this household in [insert appropriate month/year] through [insert appropriate month/year] (between approximately 18 and 24 months old), and ask you a few questions about each of them. Although I know it may be difficult, please include any children during this time who passed away.*

**Section A. Children and Caregivers**

| **Q#** | **Question** | | | | **Response** | | | | |  |
| --- | --- | --- | --- | --- | --- | --- | --- | --- | --- | --- |
| 1 | How many children in this household were born between _______ and _______ (18- 24 months old)? *Insert months/yrs when study initiated* | | | | ____ children | | | | | |
| *Please use the codes in the box below to fill out the columns. Start with the oldest child.* ***Note that if more than one child’s information is completed here****,* ***the interviewer should be automatically prompted with a new form to be completed for child 2, child 3 and any other children.*** | | | | | | | | | | |
|  | Child  *Select N/A if no 2^nd^ or 3^rd^ child* | Is CHILD a twin/ triplet?  **Yes=1**  **No=0** | Is CHILD a boy or girl?  **Boy=1**  **Girl=2** | Are you CHILD's biological mother?  **Yes=1**  **No=0** | | If no, where is CHILD's biological mother?  **Permanently lives elsewhere=1**  **Temp lives elsewhere=2**  **Died=3**  **Don’t know=87** | What date was CHILD born?  (dd/mm/yy) | Is CHILD still alive?  **Yes=1**  **No=0** | If no, when did CHILD die? (dd/mm/yy) | |
| 2a | Child 1 |  |  |  | |  | _ _/_ _ /_ _ |  | _ _/_ _ /_ _ | |
| 2b. If the child is a twin or triplet, indicate if this is the first, second, or third interview by the caregiver:   - (1) 1^st^ interview – should proceed to full set of questions - (2) 2^nd^ interview – *skip to Q28* - (3) 3^rd^ interview – *skip to Q28* - (99) N/A – Child is singleton | | | | | | | | | | |

*If multiple children, start with Child 1, simply referred to as CHILD below. Questions #3-10 should be completed by the non-maternal caregiver. If the biological mother of CHILD 1 is being interviewed, skip to Section B.*

| 3 | If not *CHILD*’s biological mother, indicate the gender of the participant: | - (1) Male - (2) Female |
| --- | --- | --- |
| 4 | If not *CHILD*’s biological mother, what is your relationship to the child? | - (1) Father - (2) Sibling - (3) Step-parent - (4) Grandparent - (5) Other relative, e.g., aunt, uncle, cousin, etc. - (6) Friend/Neighbor - (88) Other, specify: ______________________ |
| 5 | For how long has the child been under your care? | ______ years *(if less than 1, enter 0)* |
| 6 | How old are you? | __ __ years |
| 7 | What is the highest level of education that you have attended? | - (1) None - (2) Primary - (3) Secondary - (4) High School - (5) Tertiary |
| 8 | What is your current marital status? | - (1) Married - (2) Co-habitation - (3) Separated/divorced - (4) Widowed - (5) Never married |
| 9 | How many people live in this household? (include all children, adults, and yourself) | _______ people |
| 10 | How long have you lived in this community? (The community that we are in right now) | ______ years *(if less than 1, enter 0)* |

**Section B. Household Characteristics and Demographics**

*This section should be completed by the biological mother about herself or the non-maternal caregiver about the biological mother to the best of his or her knowledge.*

| **Q#** | **Question** | **Response** |
| --- | --- | --- |
| 11 | How old are you?/ How old is the mother? | - __ __ years - (87) Don’t know |
| 12 | What is the highest level of education that you (mother) have attended? | - (1) None - (2) Primary - (3) Secondary - (4) High School - (5) Tertiary - (87) Don’t know |
| 13 | What is your (mother’s) current marital status? | - (1) Married - (2) Co-habitation - (3) Separated/divorced - (4) Widowed - (5) Never married - (87) Don’t know |
| 14 | How many people live in this household? *(include all children, adults, and yourself)* | _______ people   - (99) N/A (for care givers) |
| 15 | How long have you lived in this village? *(The village that we are in right now)* | ______ years *(if less than 1, enter 0)*   - (99) N/A (for care givers) |

**Section C. Pregnancies and Children** *If non-maternal caregiver (NOT biological mother), skip to section D.*

| **Q#** | **Question** | **Response** |
| --- | --- | --- |
| 16 | How many times have you been pregnant? | ____ times |
| 17 | How many times have you given birth? | ____ births |
| 18 | How many of your children are alive today? | ____ children |
| 19 | Are you currently pregnant? | - (1) Yes - (0) No - (87) Don’t know - (99) N/A |

**Section D. HIV Testing and Counseling History**

*Non-maternal caregivers should be asked the questions in this section and answer to the best of their ability.*

| **Q#** | **Question** | **Response** |
| --- | --- | --- |
| 20 | Did you (mother) receive an HIV test when you (mother) were pregnant with CHILD? | - (1) Yes – *if yes, skip to 22* - (0) No - (87) Don’t know *– only select this response if non-maternal caregiver, skip to Section E* |
| 21 | **If no,** why not?  *Answer question, then skip to Q23.* | - (1) Did not attend ANC - (2) Was not offered a test in ANC or maternity - (3) Refused the test - (4) Already knew HIV status - (88) Other, specify: _____________________ - (87) Don’t know |
| 22 | **If yes,** what was the result of the HIV test? | - (1) HIV-positive - (2) HIV-negative - (3) Indeterminate - (87) Don’t know, specify why: _______________________ - (99) N/A |
| 23 | What was the month and year of your (mother’s) last HIV test? | - (1) Date available: __ __ /__ __ __ __ *(mm/yyyy)* - (87) Don’t know - (99) N/A (Never had an HIV test) |
| 24 | Where did you (mother) receive the last HIV test? | - (1) At ANC during pregnancy with (index) CHILD *- if 1, skip to 26* - (2) At ANC during another pregnancy - (3) During PNC - (4) Community outreach/mobile clinic - (5) VCT - (88) Other, specify: ________________ - (87) Don’t know |
| 25 | What was the result of this test? | - (1) HIV-positive - (2) HIV-negative - (3) Indeterminate - (87) Don’t know, specify why: _______________________ |
| 26 | Have you (mother) told anyone the results of your HIV test? | - (1) Yes - (0) No *– if 0, skip to Section E* - (87) Don’t know – *only select this response if non-maternal caregiver, skip to Section E* |
| 27 | **If yes,** who did you (mother) tell?  *(select all that apply)* | - (1) Partner/spouse - (2) Children - (3) Other family member - (4) Friend(s)/neighbor(s) - (5) Religious leader - (6) Support group member - (88) Other, specify: _______________________ |

**Section E. HIV Testing during this Survey**

| ***Q#*** | ***Question*** | ***Response*** |
| --- | --- | --- |
| 28 | Indicate the mother’s test result from today: | - (1) HIV-positive - (2) HIV-negative *– if 2, skip to Q30* - (3) Indeterminate - (4) Mother had documentation of HIV-positive status - (5) Mother had documentation of HIV-negative status - *if 5, skip to Section F* - (6) Mother refused - (99) N/A (non-maternal caregiver or if 2^nd^/3^rd^ interview for twin or triplet) |
| 29 | Indicate the child’s test result from today: | - (1) HIV-positive - (2) HIV-negative - (3) Indeterminate - (4) Child had documentation of HIV-positive status - (99) N/A (e.g., child deceased, refused child testing) |
| 30 | Was a DBS for the mother taken? | - (1) Yes *- if 1, skip to Q32* - (0) No - (99) N/A (if non-maternal caregiver or 2^nd^/3^rd^ interview for twin or triplet)*, skip to Q32* |
| 31 | **If no,** why? | - (1) Mother/caregiver refused - (2) Failed to get specimen - (3) Ran out of test kits - (88) Other, specify: ________________ |
| 32 | Was a DBS for child taken? | - (1) Yes - *if 1, skip to Section F* - (0) No - (99) N/A (e.g., child deceased, refused child testing) – *if 99, skip to Section F* |
| 33 | **If no,** why? | - (1) Mother/caregiver refused - (2) Failed to get specimen - (3) Ran out of test kits - (88) Other, specify: ________________ |

**Section F. Antenatal Care**

*Non-maternal caregivers should be asked the questions in this section and answer to the best of their ability.*

| **Q#** | **Question** | **Response** |
| --- | --- | --- |
| 34 | When you (mother) were pregnant with CHILD, did you attend antenatal care? | - (1) Yes - (0) No   Specify reason: ___________________   - *If 0, skip to Section G* - (87) Don’t know *– if 87, skip to Section G* |
| 35 | **If yes,** did you (mother) receive antenatal care for CHILD in the facility in this catchment area?  *(identify the facility for the participant)* | - (1) Yes, received care at facility in catchment area only *– if 1, skip to Q37* - (2) Yes, received care at facility in catchment area and other facilities - (0) No - (87) Don’t know *– if 87, skip to Q37* |
| 36 | **If you answered “No” or “Received care at multiple facilities,”** why?  *(select all that apply)* | - (1) Did not live in this area while pregnant - (2) Temporarily moved to home/partners’ home village due to the pregnancy - (3) Temporarily moved because of her/her partners’ school or work - (4) Did not like the catchment area facility   Specify reason: ____________________________   - (88) Other, specify: ______________________ - (87) Don’t know |
| 37 | How did you (mother) travel to the facility for antenatal care?  *(select all that apply)* | - (1) Foot - (2) Bicycle - (3) Motorbike - (4) Public transport - (5) Pony - (6) Hired Taxi - (7) Own car - (8) Borrowed car - (99) N/A - (87) Don’t know |
| 38 | How much did transportation (round trip) cost to get to the facility and back to where you (mother) live/were staying? | - (1) _____ Maloti *(enter 0 if no cost)* - (87) Don’t know |
| 39 | How many weeks/months pregnant with CHILD were you (mother) when you FIRST received ANC?  *(select only one)* | - (1) Number in gestational weeks: ___ ___ - (2) Number in months: ___ ___ - (87) Don’t know |
| 40 | How many ANC visits did you (mother) attend when you (mother) were pregnant with CHILD? | - (1) Visits known: _______ visits - (87) Don’t know |
| 41 | During CHILD’s pregnancy, did you (mother) receive a Mother-Baby Pack (MBP)? | - (1) Yes - (0) No - (87) Don’t know |

**Section G. Delivery**

*Non-maternal caregivers should be asked the questions in this section and answer to the best of their ability.*

| **Q#** | **Question** | **Response** |
| --- | --- | --- |
| 42 | Was CHILD delivered in the facility in this catchment area? *(Name facility)* | - (1) Yes *-if 1, skip to Q45* - (0) No - (87) Don’t know – *if 87, skip to Q46* - (99) N/A (No delivery services in catchment area) |
| 43 | **If CHILD was not delivered in facility in catchment area**, where did CHILD’s delivery take place? | - (1) Your (mother) home - (2) Other home - (3) Other hospital, specify: __________________________ - (4) Other health center, specify: ______________________ - (88) Other, specify: ________________________________ - (87) Don’t know |
| 44 | **If CHILD was not delivered in facility in catchment area**, why did you (mother) deliver at this place?  *(select all that apply)* | - (1) Lack of transport - (2) Lack of money for transport and/or services - (3) Preferred by husband - (4) Preferred by other family member - (5) Do not see a reason to deliver in a facility - (6) Early/short labor - (7) Negative health care worker attitude at facility - (8) Custom to deliver in home village - (9) Better services at facility she delivered in - (10) No maternity services in catchment facility - (88) Other, specify: _________________________________ - (87) Don’t know |
| 45 | How did you (mother) travel to the facility for delivery?  *(select all that apply)* | - (1) Foot - (2) Bicycle - (3) Motorbike - (4) Public transport - (5) Pony - (6) Hired Taxi - (7) Own car - (8) Borrowed car - (87) Don’t know - (88) Other, specify: _________________________________ - (99) N/A |
| 46 | Was CHILD delivered by cesarean section? | - (1) Yes, emergency - (2) Yes, scheduled - (0) No - (87) Don’t know |
| 47 | Did you (mother) experience any complications during delivery or as a result of delivery?  *(select all that apply)* | - (1) Excessive blood loss following birth (post-partum hemorrhage - (2) High fever following birth (puerperal sepsis) - (3) Seizures (eclampsia) - (4) Inflammation and premature rupture of membranes (chorioamnionitis) - (5) Baby not positioned normally (malpresentation) - (6) Fetal distress (e.g., breathing problems) - (7) None - (88) Other, specify: ______________________________ - (87) Don’t know |
| 48 | Did it cost money for CHILD’s delivery services? | - (1) Yes - (0) No *– if 0, skip to Q50* - (87) Don’t know*– if 87, skip to Q50* |
| 49 | **If** **yes**, how much was paid for delivery services? | - (1) Amount: __________________ (in Maloti) - (87) Don’t know |
| 50 | What was the estimated gestational age (preferred) OR estimated date of delivery?  *(select only 1 option and write in appropriate response)* | - (1) _______ gestational weeks - (2) EDD: __ __/__ __/__ __ __ __ *(dd/mm/yyyy)* - (87) Don’t know |
| 51 | Did you (mother) have to stay extra days in the facility for any reason? | - (1) Yes, mother required prolonged stay Reason:____________________________ - (0) No - (99) N/A (CHILD was not delivered in facility) |
| **Child Delivery Information**  ***If child is a twin or triplet and the first interview has already been conducted, the interview should be started here.*** | | |
| 52 | Was CHILD born alive or dead? | - (1) Alive - (2) Dead*– skip to Section K* |
| 53 | Was CHILD born, early, late or about on time? | - (1) Pre-term (early) - (2) Term (on time) - (3) Post-term (late) - (87) Don’t know |
| 54 | Were you (or mother) told CHILD had a low birth weight? | - (1) Yes - (0) No - (87) Don’t know |
| 55 | What was CHILD’s birth weight? | - (1) Weight known: ______ kg - (87) Don’t know |
| 56 | Did the CHILD have to stay extra days in the facility for any reason? | - (1) Yes, CHILD required prolonged stay   Reason: _____________________________   - (0) No - (99) N/A (CHILD was not delivered in facility) |

**Section H. Child Death**

*Only ask the following questions to the mother/caregiver if the child has died. If child is still alive, mark Yes to Q57, then skip to Section I. Postpartum Care.*

| 57 | Is child alive? | - (1) Yes – *skip to Section I* - (0) No |
| --- | --- | --- |
| 58 | **If no,** what was CHILD’s primary cause of death?  *(Stillbirths should be captured on Q52)* | - (1) Diarrhea (gastroenteritis) - (2) Pneumonia (acute respiratory infection) - (3) Measles - (4) Malaria - (5) Birth defect (congenital anomalies) - (6) Lack of oxygen around the time of birth (asphyxia) - (7) Neonatal tetanus - (8) Malnutrition - (9) Meningitis - (10) Tuberculosis - (11) Medication side effect, specify: _________________________________ - (12) Accidents (traffic, etc) - (13) Violence - (14) No cause given/determined - (88) Other, specify: ________________________________________ - (87) Don’t know |
| 59 | Was CHILD receiving medical care at the time of death? | - (1) Yes - (0) No - *if 0, skip to Q61* - (87) Don’t know *– if 87, skip to Q61* |
| 60 | **If yes,** where was treatment sought? | - (1) Had received care from a hospital - (2) Had received care at a health center - (3) Had received care from a traditional healer - (4) Had received care from a pharmacy - (5) Had not sought any care for this illness - (87) Don’t know |
| 61 | Where did the death occur? | - (1) At home - (2) At someone else’s home - (3) Traditional healer - (4) Traffic accident - (5) In transit to care - (6) At health center - (7) At hospital - (88) Other, specify: __________________________ - (87) Don’t know |
| 61a | Did child die within two weeks of life? | - (1) Yes – *if yes, skip to Section K* - (0) No |

**Section I. Postpartum Care**

*Non-maternal caregivers should be asked the questions in this section and answer to the best of their ability.*

| **Q#** | **Question** | **Response** |
| --- | --- | --- |
| 62 | When was CHILD first taken to a facility after delivery? | - (1) Within 3 days of birth - (2) Within 1 week after birth - (3) Approximately 6-8 weeks after birth - (4) Approximately 10 weeks after birth - (5) Approximately 14 weeks after birth - (6) After 14 weeks postpartum - (7) Never – *if 7, skip to Q64* - (87) Don’t know |
| 63 | When was CHILD’s second visit to a facility after birth? | - (1) Within 1 week after birth - (2) Approximately 6-8 weeks after birth - (3) Approximately 10 weeks after birth - (4) Approximately 14 weeks after birth - (5) After 14 weeks postpartum - (6) Never - (87) Don’t know |
| *The next several questions address immunizations that CHILD has received. You may want to use a vaccination visit schedule or other visual aid to help remind them.* | | |
| 64 | Did CHILD receive the BCG vaccination at birth? | - (1) Yes - (2) No, but received after birth - (0) No, never received - (87) Don’t know |
| 65 | What Oral Polio Vaccine (OPV) did the child receive?  (*select all that apply)* | - (1) OPV 0 - (2) OPV 1 - (3) OPV 2 - (4) OPV 3 - (5) None - (87) Don’t know |
| 66 | What Pentavalent did CHILD receive?  *If needed, explain Penta includes diphtheria, tetanus, pertussis, hepatitis B surface antigen and Hib conjugate*  (*select all that apply)* | - (1) Penta 1 - (2) Penta 2 - (3) Penta 3 - (4) None - (87) Don’t know |
| 67 | What Measles vaccination did CHILD receive?  (*select all that apply)* | - (1) Measles 1 - (2) Measles 2 - (3) None - (87) Don’t know |
| 68 | Did CHILD receive the Tetanus (DT) vaccination?  *Typically at 18 months of age* | - (1) Yes - (0) No - (87) Don’t know |
| 69 | Has CHILD ever spent the night in a clinic or hospital AFTER being discharged from birth facility? | - (1) Yes - (0) No *– if 0, skip to Q72* - (87) Don’t know*– if 87, skip to Q72* |
| 70 | How many times has CHILD been hospitalized? | - (1) 1 time - (2) 2 times - (3) 3 times - (4) 4 times - (5) More than 4 times - (87) Don’t know |
| 71 | What were the reasons for the hospitalization(s)?  For each hospitalization, select the box and write in the reason. If for one or more hospitalizations, the reasons are unknown, select the box and write in ‘don’t know.’ | - Hospitalization 1: ­­­­­­­­­__________________________ - Hospitalization 2: _________________________ - Hospitalization 3: _________________________ - (87) Don’t know - (99) N/A |
| 72 | Has a health worker ever told you (or mother) that CHILD has growth problems? | - (1) Yes - (0) No – *if 0, skip to Section J* - (87) Don’t know - *if 87, skip to Section J* |
| 73 | **If yes,** at what age(s) did CHILD experience growth problems?  *(select all that apply)* | - (1) 0-6 months of age - (2) 7-12 months of age - (3) 13-18 months of age - (4) 19-24 months of age - (87) Don’t know |

**Section J. Infant Feeding**

*Non-maternal caregivers should be asked the questions in this section and answer to the best of their ability.*

| **Q#** | **Question** | **Response** |
| --- | --- | --- |
| 74 | Was CHILD ever breastfed? | - (1) Yes - (0) No, why not? ________________________ - *If 0, skip to Section K* - (87) Don’t know - *If 87, skip to Section K* |
| 75 | Who breastfed CHILD?  *(select all that apply)* | - (1) Biological mother - (2) Someone else, specify: ___________________________ - (87) Don’t know |
| 76 | Is CHILD still receiving any breast milk? | - (1) Yes - *if 1, skip to Q78* - (0) No |
| 77 | At what age did the baby stop receiving any breast milk? | - (1) Age known: ____ months - (87) Don’t know |
| 78 | At what age were any other liquids or foods introduced (even if still breastfeeding)? | - (1) Age known: ____ months of age - (87) Don’t know |

**Section K. Maternal Health**

*Non-maternal caregivers should be asked the questions in this section and answer to the best of their ability.*

| **Q#** | **Question** | **Response** |
| --- | --- | --- |
| 79 | Have you (mother) experienced or been diagnosed with any of the following in the last 3 years?  *(read all responses, select all that apply)* | - (0) None - (1) High blood pressure (hypertension) - (2) Tuberculosis - (3) Lower respiratory disease/pneumonia - (4) Significant weight loss/malnutrition - (5) Malaria - (6) Hepatitis - (7) Kidney disease - (87) Don’t know |
| 80 | Have you (mother) been told to go to the hospital for any reason other than childbirth in the last 3 years? | - (1) Yes, hospitalized - (2) Yes, not hospitalized*– if 2, skip to Q83* - (0) No *– if 0, skip to Q83* - (87) Don’t know *– if 87, skip to Q83* |
| 81 | **If yes,** how many times were you hospitalized? | - (1 1 time - (2) 2 times - (3) 3 times - (4) 4 times - (5) More than 4 times - (87) Don’t know |
| 82 | What was the reason for the hospitalizations?  *Select all that apply. Write in or enter as many hospitalizations as known. Select ‘don’t know’ if there is at least one hospitalization for which the reason is unknown.* | - (1) Hospitalization 1: ­­­­­­­­­________________________ - (2) Hospitalization 2: ________________________ - (3) Hospitalization 3: ________________________ - (87) Don’t know |
| 83 | Have you (mother) received a blood transfusion in the last 3 years? | - (1) Yes - (0) No - (87) Don’t know |
| *Only ask the following questions to the caregiver if the mother has died. Otherwise, skip to the end of this section.* | | |
| 84 | What was the date of her death? | __ __ / __ __ /__ __ __ __ *(dd/mm/yyyy)*  *Include as much of the date as possible, such as month and year even if day is unknown* |
| 85 | What was her pregnancy status at the time of death? | - (1) Not pregnant - (2) Pregnant - (3) In labor and delivery - (4) Shortly after delivery (within 1 week) - (87) Don’t know |
| 86 | What was the primary cause of death? | - (1) Excessive blood loss (hemorrhage) - (2) High fever following birth (puerperal sepsis) - (3) High blood pressure (hypertension) - (4) Tuberculosis - (5) Lower respiratory disease/pneumonia - (6) Diarrhea/dehydration - (7) Significant weight loss (wasting/malnutrition) - (8) Malaria - (9) Hepatitis - (10) Kidney disease - (11) Medication side effect, specify: _________________________________ - (12) Accidents (traffic, etc.) - (13) Violence - (14) No cause given/determined - (88) Other, specify: ________________________________________ - (87) Don’t know |
| 87 | Was she receiving medical care at the time of death? | - (1) Yes - (0) No *– if 0, skip to Q89* - (87) Don’t know*– if 87, skip to Q89* |
| 88 | **If yes,** where was treatment sought? | - (1) Had received care from a hospital - (2) Had received care at a health center - (3) Had received care from a traditional healer - (4) Had received care from a pharmacy - (87) Don’t know - (99) N/A |
| 89 | Where did the death occur? | - (1) At her home - (2) At someone else’s home - (3) Traditional healer - (4) Traffic accident - (5) In transit to care - (6) At health center - (7) At hospital - (88) Other, specify: __________________________ - (87) Don’t know |

***If biological mother of the child is HIV-negative, or if she tested HIV-positive on the day of the interview, thank the participant and end the interview. Do you want to continue with the interview?***

**Section L. HIV Care**

*Only for mothers who had a known HIV-positive status before the interview was initiated. Those who tested HIV-positive on the day of the interview should not respond to the following questions. Non-maternal caregivers of children with an HIV-positive mother should be asked the questions in this section and answer to the best of their ability.*

| **During Antenatal Care** | | |
| --- | --- | --- |
| 90 | During CHILD’s pregnancy, did you (mother) have a CD4 test? | - (1) Yes - (0) No *– If 0, skip to Q92* - (87) Don’t know*– If 87, skip to Q92* - (99) N/A*– If 99, skip to Q92* |
| 91 | **If yes,** what was the result? *(document from record)* | - (1) CD4 count: ______________ per cu mm - (87) Unknown (no result available) |
| 92 | During CHILD’s pregnancy, were you (mother) given any drugs to decrease the chance of passing HIV to the CHILD (either in the MBP or by themselves)? | - (1) Yes – *If 1, skip to Q94* - (0) No - (87) Can’t remember/don’t know *– if 87, skip to Q96* - (99) N/A (was not HIV-positive at the time) *– if 99, skip to Q96* |
| 93 | **If you (mother) were not given any HIV medications,** why not?  *Answer Q, then skip to Q96.* | - (1) Did not attend ANC - (2) Was not offered medications in ANC - (3) Refused medications - (88) Other, specify: _________________________ |
| 94 | What HIV medications were you (mother) given during CHILD’s pregnancy? | - (1) AZT alone to take every day during pregnancy - (2) AZT first then switched to ART drugs that I was told to take for life - (3) ART drugs that I was told to take for life *– if 3, skip to Q96* - (4) None *– if 4, skip to Q96* - (87) Can’t remember/don’t know *– if 87, skip to Q96* - (88) Other, specify: _________________________________ |
| 95 | During CHILD's pregnancy, what month and year did you (mother) start taking the AZT tablets? | - (1) Date available: __ __ /__ __ __ __ *(mm/yyyy)* - (2) Received them but never took them - (87) Can’t remember/don’t know - (99) N/A |
| 96 | When did you (mother) start taking ART? | - (1) Before pregnancy with CHILD - (2) During pregnancy with CHILD - (3) During breastfeeding of CHILD - (4) After breastfeeding of CHILD - (87) Can’t remember/don’t know *– if 87, skip to Q100* - (99) N/A (not on ART) *– if 99, skip to Q100* |
| 97 | What was the month and year you (mother) started ART? | - (1) Date available: __ __ /__ __ __ __ *(mm/yyyy)* - (87) Can’t remember/don’t know |
| 98 | What ART regimen were you (mother) taking during pregnancy with CHILD? | - (1) TDF+3TC+EFV (1f) - (2) TDF+3TC+NVP(1e) - (3) AZT+3TC+EFV (1d) - (4) AZT+3TC+NVP(1c) - (88) Other, specify: _________________________ - (87) Can’t remember/don’t know - (99) N/A |
| 99 | What ART regimen are you (mother) currently taking? | - (1) TDF+3TC+EFV - (2) TDF+3TC+NVP - (3) AZT+3TC+EFV - (4) AZT+3TC+NVP - (88) Other, specify: _________________________ - (87) Don’t know - (99) N/A |
| **During Labor and Delivery** | | |
| 100 | What HIV drugs did you (mother) take during labor?  *(select all that apply)* | - (1) Continued ART regimen - (2) AZT - (3) NVP - (4) Combivir (AZT/3TC) - (5) None - (87) Don’t know/cannot remember - (99) N/A (not known positive at the time) |
| **During Postpartum Care** | | |
| 101 | Did you (mother) take ARV prophylaxis in the week after CHILD’s delivery (AZT/3TC postpartum ‘tail’)? | - (1) Yes - (0) No - (87) Don’t know - (99) N/A (on ART or not known positive at the time) |
| 101a | Did CHILD died at birth? | - (1) Yes – if 1, skip to Section N - (0) No |
| 102 | Was CHILD given the first dose of Nevirapine syrup within 3 days after delivery? | - (1) Yes, given by the health worker before discharge after facility delivery - (2) Yes, given at home by mother - (3) Yes, given at home by community/village health worker - (4) Yes, given at home by someone else - (0) No - (87) Don’t know - (99) N/A (not known positive at the time) |
| 103 | How long was CHILD given infant Nevirapine syrup? | - (1) Never *– if 1, skip to Q105* - (2) 6 weeks - (3) Throughout the entire breastfeeding period *(select this response if still or stopped BF)* - (4) Until mother was told to stop by health worker while still breastfeeding - (5) Until mother chose to stop while still breastfeeding - (87) Don’t know |
| 104 | What month and year did the child stop receiving NVP?  *Include date if available for (1) and (2)* | - (1) Until the end of breastfeeding   Date available: __ __ /__ __ __ __ (mm/yyyy)  *– if 1, skip to Q106*   - (2) Before the end of breastfeeding   Date available: __ __ /__ __ __ __ (mm/yyyy)   - (3) Still receiving NVP   *– if 3, skip to Q106*   - (87) Don’t know   *– if 87, skip to Q106*   - (99) N/A (Child never given NVP) |
| 105 | **If never (99), or if baby did not receive NVP throughout breastfeeding (2),** why?  *Mark all that apply.* | - (1) Mother on ART - (2) Did not want to - (3) Partner/family member did not want me to - (4) Could not travel to clinic regularly to pick up drugs - (5) Child experienced side effects - (6) Did not yet know HIV status - (7) Child was HIV-positive and began treatment - (8) Didn’t know I was supposed to - (9) Never given any more medicine after MBP - (88) Other, specify: ________________________________ - (87) Don’t know |
| 106 | Was CHILD given cotrimoxazole (CTX) to take every day beginning at 6 weeks?  *Provide clear guidance on distinguishing CTX from NVP for women.* | - (1) Yes - (0) No *– if 0, skip to Q108* - (87) Don’t know |
| 107 | When was the last pick up of CTX for CHILD? | - (1) Within the last month - (2) Within the last 2-3 months - (3) Within the last 4-6 months - (4) More than 6 months ago - (5) Child negative – no longer receiving CTX - (6) Have never picked up/given child CTX - (87) Don’t know |
| 108 | Was CHILD ever tested for HIV? | - (1) Yes - (0) No *- if 0, skip to Q114* - (87) Don’t know |
| 109 | In what month and year was CHILD first tested for HIV? | - (1) Date available: __ __ /__ __ __ __ (mm/yyyy) - (87) Don’t know - (99) N/A (Never tested or don’t know if tested) |
| 110 | What was the result of CHILD's first HIV test? | - (1) HIV-positive - (2) HIV-negative - (3) Indeterminate - (4) Did not receive result   Why not? ______________________________________   - (87) Don’t know |
| 111 | Did CHILD receive any other HIV tests? | - (1) Yes - (0) No - (87) Don’t know |
| 112 | In what month and year was CHILD’s last test for HIV? | - (1) Date available:__ __ /__ __ __ __ (mm/yyyy) - (87) Don’t know - (99) N/A (Did not receive other tests or don’t know if received other testslth careme)nd (2)gt area)) – if 99, skip to Q114. Should have this skip here but it wasn’t added from the beginning so decided to keep it out. If participant doesn’t know about the child’s tests, should indicate don’t know for Q113. |
| 113 | What was the result of CHILD's last test for HIV? | - (1) HIV-positive - (2) HIV-negative - (3) Indeterminate - (4) Did not receive result   Why not? ______________________________________   - (87) Don’t know |
| 114 | For women on ART only: Did you (mother) receive HIV treatment for the entire time that you were breastfeeding CHILD? | - (1) Yes *- if 1, skip to Q116* - (0) No - (87) Don’t know *- if 87, skip to Q116* - (99) N/A *-if 99, skip to Q116 (not on ART)* |
| 115 | If no, why not?  *Mark all that apply.* | - (1) Was only given prophylaxis for pregnancy/delivery - (2) Did not want to - (3) Partner/family member did not want me to - (4) Could not travel to clinic regularly to pick up drugs - (5) Experienced side effects - (6) Did not yet know HIV status - (88) Other, specify: _________________________________ - (87) Don’t know |

**Section M. Child Treatment and Adherence**

*Only complete this section if child is known HIV-positive.* *If child is not positive, select N/A for Q116, then skip to the end of the section. Non-maternal* *caregivers should be asked the questions in this section and answer to the best of their ability.*

| **Q#** | **Question** | **Response** |
| --- | --- | --- |
| 116 | Was CHILD initiated on ART? | - (1) Yes - (0) No, why? ______________________________ - *If 0, skip to the end of the section* - (99) N/A *– if 99, skip to the end of the section* |
| 117 | What date was CHILD initiated on ART? | - (1) Date known: __ __ /__ __ __ __ *(mm/yyyy)* - (87) Don’t know - (99) N/A |
| 118 | What ART regimen is CHILD currently taking?  (LPV/r =Kaletra) | - (1) ABC+ 3TC + LPV/r - (2) AZT + 3TC + LPV/r - (3) ABC + 3TC + NVP - (4) AZT + 3TC + NVP   (or Zidolam-n or Combivir + NVP)   - (88) Other, specify: _________________________ - (87) Unknown - (99) N/A |
| 119 | How many drug doses does CHILD take each day? | - (1) One (OD) - (2) Two (BD) - (88) Other, specify: _____ - (99) N/A |
| 120 | How many doses has CHILD missed in the last 3 days? | - (1) One - (2) Two - (3) Three - (4) Four - (5) Five - (6) Six - (7) None - (88) Other, specify: ______ - (99) N/A |

**Section N. Maternal Adherence**

*Non-maternal caregivers should skip this section and end interview here.*

| **Q#** | **Question** | **Response** |
| --- | --- | --- |
| 121 | Visual Analog Scale: *Instruct the respondent to do the following:*  Point to the number on the line at the point showing your best guess about how much you took your **ARV or ART pills** during pregnancy. “0” means you took none of the pills you were supposed to. “5” means you took about half of the pills as instructed. “10” means you took every single dose as instructed. | *Write the number that the respondent indicates in the response column:*  ____________________     - (99) N/A   *Select N/A if mother did not learn her HIV status during index pregnancy (see response to Q22)* |
|  | **0 1 2 3 4 5 6 7 8 9 10**  **I____I____I____I____I____I____I____I____I____I____I** | |
| 122 | Visual Analog Scale: *Instruct the respondent to do the following IF woman was on* ***ART during CHILD pregnancy****:*  Point to the number on the line at the point showing your best guess about how much you took your **ART pills** throughout breastfeeding. “0” means you took none of the pills you were supposed to. “5” means you took about half of the pills as instructed. “10” means you took every single dose as instructed. | *Write the number that the respondent indicates in the response column:*  ____________________     - (99) N/A   *Select N/A if mother was on prophylaxis during pregnancy or if CHILD died at birth. If the mother switched to ART during breastfeeding, ask this question about her adherence to ART*. |
| **0 1 2 3 4 5 6 7 8 9 10**  **I____I____I____I____I____I____I____I____I____I____I** | | |
| 123 | Visual Analog Scale: *Instruct the respondent to do the following IF woman was on* ***prophylaxis during CHILD pregnancy****:*  Point to the number on the line at the point showing your best guess about how much **NVP syrup** you gave CHILD **during the entire breastfeeding period**. “0” means you never gave the syrup. “5” means you gave it about half of the time as instructed. “10” means you gave every single dose as instructed. | *Write the number that the respondent indicates in the response column:*  ____________________     - (99) N/A   *Select N/A if the mother was on ART when breastfeeding the child or if CHILD died at birth. If the mother switched to ART during the breastfeeding period, you can ask her about the NVP syrup given to the baby before she started ART.* |
| **0 1 2 3 4 5 6 7 8 9 10**  **I____I____I____I____I____I____I____I____I____I____I** | | |
| 124 | Visual Analog Scale: *Instruct the respondent to do the following IF woman was on* ***ART during CHILD pregnancy****:*  Point to the number on the line at the point showing your best guess about how much **NVP syrup** you gave CHILD **during the first 6 weeks of life**. “0” means you never gave the syrup. “5” means you gave it about half of the time as instructed. “10” means you gave every single dose as instructed. | *Write the number that the respondent indicates in the response column:*  ____________________     - (99) N/A   *Select N/A if the mother was on prophylaxis during index pregnancy or if CHILD died at birth.* |
| **0 1 2 3 4 5 6 7 8 9 10**  **I____I____I____I____I____I____I____I____I____I____I** | | |
| 125 | **If currently on ART,** how many drug doses do you take each day? | - (1) One - (2) Two - (88) Other, specify: _____ - (99) N/A |
| 126 | **If currently on ART,** how many doses have you missed in the last 3 days? | - (1) One - (2) Two - (3) Three - (4) Four - (5) Five - (6) Six - (7) None - (88) Other, specify: ______ - (99) N/A |
| 127 | When was your (mother) last visit to the ART clinic? | __ __ / __ __ /__ __ __ __ *(dd/mm/yyyy)*  *Include as much of the date as possible, even if day is unknown* |
| 128 | Where did you and your child get your postnatal care in first 2 years? | - (1) Together in MCH clinic on same day - (2) Both in MCH clinic but on different days - (3) In different clinics on same day - (4) In different clinics on different days |
| 129 | Where do you and your child get your care currently? | - (1) Together in MCH clinic on same day - (2) Both in MCH clinic but on different days - (3) In different clinics on same day - (4) In different clinics on different days |

**Thank you for your time. We have completed the interview.**

**Please use the space below to write in any comments from the interview:**
